# Prolonged prone positioning under VV-ECMO is safe and improves oxygenation and respiratory compliance

**DOI:** 10.1186/s13613-015-0078-4

**Published:** 2015-11-04

**Authors:** Antoine Kimmoun, Sylvain Roche, Céline Bridey, Fabrice Vanhuyse, Renaud Fay, Nicolas Girerd, Damien Mandry, Bruno Levy

**Affiliations:** CHU Nancy, Service de Réanimation Médicale Brabois, Pole Cardiovasculaire et Réanimation Médicale, Hôpital Brabois, 54511 Vandoeuvre les Nancy, France; INSERM U 1116, Groupe Choc, Equipe 2, Faculté de Médecine, 54511 Vandoeuvre les Nancy, France; Université de Lorraine, 54000 Nancy, France; CHU Nancy, Département de Radiologie, Hôpital Brabois, 54511 Vandoeuvre les Nancy, France; CHU Nancy, Département de Chirugie Cardiaque Brabois, Pole Cardiovasculaire et Réanimation Médicale, Hôpital Brabois, 54511 Vandoeuvre les Nancy, France; INSERM, Centre d’Investigations Cliniques-9501 and CHU de Nancy, 54511 Vandoeuvre les Nancy, France

**Keywords:** ARDS, ECMO, Prone positioning

## Abstract

**Background:**

Data are sparse regarding the effects of prolonged prone positioning (PP) during VV-ECMO. Previous studies, using short sessions (<12 h), failed to find any effects on respiratory system compliance. In the present analysis, the effects of prolonged PP sessions (24 h) were retrospectively studied with regard to safety data, oxygenation and respiratory system compliance.

**Methods:**

Retrospective review of 17 consecutive patients who required both VV-ECMO and prone positioning. PP under VV-ECMO was considered when the patient presented at least one unsuccessful ECMO weaning attempt after day 7 or refractory hypoxemia combined or not with persistent high plateau pressure. PP sessions had a duration of 24 h with fixed ECMO and respiratory settings. PP was not performed in patients under vasopressor treatment and in cases of recent open chest cardiac surgery.

**Results:**

Despite optimized protective mechanical ventilation and other adjuvant treatment (i.e. PP, inhaled nitric oxide, recruitment maneuvers), 44 patients received VV-ECMO during the study period for refractory acute respiratory distress syndrome. Global survival rate was 66 %. Among the latter, 17 patients underwent PP during VV-ECMO for a total of 27 sessions. After 24 h in prone position, PaO_2_/FiO_2_ ratio significantly increased from 111 (84–128) to 173 (120–203) mmHg (*p* < 0.0001) while respiratory system compliance increased from 18 (12–36) to 32 (15–36) ml/cmH_2_O (*p* < 0.0001). Twenty-four hours after the return to supine position, tidal volume was increased from 3.0 (2.2–4.0) to 3.7 (2.8–5.0) ml/kg (*p* < 0.005). PaO_2_/FiO_2_ ratio increased by over 20 % in 14/14 sessions for late sessions (≥7 days) and in 7/13 sessions for early sessions (<7 days) (*p* = 0.01). Quantitative CT scan revealed a high percentage of non-aerated or poorly-aerated lung parenchyma [52 % (41–62)] in all patients. No correlation was found between CT scan data and respiratory parameter changes. Hemodynamics did not vary and side effects were rare (one membrane thrombosis and one drop in ECMO blood flow).

**Conclusion:**

When used in combination with VV-ECMO, 24 h of prone positioning improves both oxygenation and respiratory system compliance. Moreover, our study confirms the absence of serious adverse events.

**Electronic supplementary material:**

The online version of this article (doi:10.1186/s13613-015-0078-4) contains supplementary material, which is available to authorized users.

## Background

One year after the publication of the PROSEVA study, the association of prone positioning (PP) and lung-protective ventilation has become routine management for patients with acute respiratory distress syndrome (ARDS) [1]. However, refractory ARDS is still observed in all recent observational and randomized trials [1–3]. In these cases, veno-venous extra-corporeal membrane oxygenation (VV-ECMO) is indicated while awaiting lung function recovery [4]. Indeed, in the CESAR trial, a protocol including VV-ECMO was associated with a decreased mortality rate when compared to a conventional lung protective strategy [2].

The management of persistent severe hypoxemia under VV-ECMO requires a multi-step clinical approach including the optimization of VV-ECMO blood flow, red blood cell transfusion, moderate hypothermia, optimization of native lung function, short-action beta-blockers and finally PP [5]. PP can be effective in patients with VV-ECMO given that the use of ultra-protective ventilation [i.e. 3–4 mL/kg Vt] may increase the proportion of poorly-aerated areas in dependent lung regions [6]. As a result, PP during VV-ECMO may recruit the dorsal regions of the lungs, facilitate lung drainage and therefore improve oxygenation. The value of PP during VV-ECMO has furthermore been previously described in a few studies, the largest being a study by Guervilly et al. in which 12 h of PP significantly improved the PaO_2_/FiO_2_ ratio in 15 ARDS patients on VV-ECMO after a median of 9 days [7]. Altogether, data from this and other previously published studies suggest that PP during VV-ECMO is safe when performed by a referent team and ultimately improves oxygenation. Of note, these studies failed to find any improvement in respiratory system compliance [6–11].

Since PP during VV-ECMO also carries potentially harmful effects, it is thus imperative to better delineate its effects and putative indications.

Previous studies, using short sessions (>12 h), failed to find any effects on respiratory system compliance. In light of the above, the present study was aimed at retrospectively analyzing the effects of prolonged PP sessions (24 h) on safety data, oxygenation and respiratory system compliance.

## Methods

The ECMO database of our 14-bed ICU was retrospectively reviewed to identify patients who received PP during VV-ECMO between January 2012 (first treatment in our ICU) and January 2014. The study protocol was evaluated by the local Ethics Committee (Comité de Réflexion Ethique Nanceien Hospitalo-Universitaire) which waived written informed consent due to both the retrospective study design and because PP and VV-ECMO are an integral part of care provided to patients with ARDS. All patients or their relatives were informed that some data could be used for clinical research. We included patients with severe ARDS as defined by the BERLIN consensus [12].

### ARDS management

All patients were treated in accordance with the latest recommended guidelines. In particular, treatment in ICU included the systematic use of protective ventilation, transient use of paralyzing agents, diuresis to dry weight, prone positioning, recruitment maneuvers, transient use of inhaled nitric oxide and high positive end expiratory pressure (PEEP) levels [2].

### Indication of ECMO

VV-ECMO was considered in patients with an optimal protective ventilator setting (Vt at 6 mL/kg of predicted body weight, PEEP adjusted to maintain a plateau pressure (pPlat) between 27 and 30 cmH_2_O) after the failure of at least one prone positioning session with one of the following criteria adapted from the ongoing EOLIA trial: (1) PaO_2_/FiO_2_ < 50 mmHg for more than 3 h under FiO_2_ > 80 %; (2) PaO_2_/FiO_2_ < 85 mmHg for more than 6 h; (3) pPlat > 35 cmH_2_O despite adjustment of *Vt* and PEEP; (4) pH < 7.25 for more than 6 h despite an increase in respiratory rate to 35/min. VV-ECMO was either initiated in our intensive care unit (ICU) or in another hospital. In the latter instance, all patients were transferred to our ICU immediately after instituting VV-ECMO by our mobile ECMO team.

### ECMO management

All VV-ECMOs were performed using percutaneous cannulation under echocardiography. A femoro-jugular circuit was implanted whenever possible, with femoro–femoral jugular circuit as an alternative. A servo-controlled centrifugal pump (Rotaflow console, Maquet, Hirrlingen, Germany) and poly-methyl pentene oxygenators (Quadrox Bioline oxygenator system Maquet, Hirrlingen, Germany) were used. The circuit and the oxygenator were fully coated with heparin. VV-ECMO flow was adapted daily according to cardiac output measured by echocardiography in order to maintain an ECMO blood flow/cardiac output ratio of at least 0.7 [13]. Sweep gas flow was titrated in order to maintain PaCO_2_ between 40 and 45 mmHg. Oxygen fraction delivered on the membrane (FDO_2_) was adjusted on post-oxygenator blood gas. Heparin was continuously infused to obtain an anti-Xa activity at 0.1–0.2.

### Respiratory management under VV-ECMO

All patients were ventilated in volume control mode. Ultraprotective ventilation was applied during the first 48 h with the following settings: Vt at 1.5–3 ml/kg of predicted body weight, respiratory rate between 8 and 12/min, PEEP between 10 and 18 adapted for a pPlat at 25 cmH_2_O. FiO_2_ was adapted for a SpO_2_ between 88 and 95 %. All patients were sedated and paralyzed by besilate cisatracurium (Hospira France, France) during the first 24 h. After 24 h, besilate cisatracurium was discontinued when possible.

### Weaning procedure

After the first 48 h, Vt was increased daily when possible, respecting a pPlat at 25 cmH_2_O. When Vt was >5 ml/kg of predicted body weight and respiratory rate >15/min, ECMO and sweep gas flow were progressively decreased respecting the following criteria: (1) PaO_2_/FIO_2_ > 150 mmHg, (2) FiO_2_ on ventilator <60 %, (3) PaCO_2_ < 50 mmHg, (4) pPlat < 25 cmH_2_O. ECMO was halted if the above criteria were respected in a non paralyzed patient after a successful 12–24 h session with a sweep gas flow at 0 L/min.

### Indication for prone positioning under VV-ECMO

Prone positioning placement was only performed in one of the two following conditions: (1) Failure of attempts to wean VV-ECMO after at least 7 days under VV-ECMO combined with the need of therapeutic sedation, (2) Refractory hypoxemia with PaO_2_/FiO_2_ ratio <85 mmHg under FiO_2_ 100 % both on the ventilator and the membrane despite optimal VV-ECMO and ventilator settings combined or not with persistent high plateau pressure (>25 cmH_2_O) despite ultra-protective ventilation. Further sessions were also performed according to the same indications.

### Contraindication for placement in prone position under VV-ECMO

Given the potential risks of PP during VV-ECMO, PP was not performed in patients under vasopressor treatment and in cases of recent open chest cardiac surgery. PP was proposed only in patients who were still under sedation and in whom it was not possible to use partial ventilatory support.

### Protocol for prone positioning under VV-ECMO (Additional files 1, 2)

The protocol was adapted from PROSEVA guidelines for prone positioning placement and has been published elsewhere [1, 6]. The detailed protocol is described in the supplementary material.

### Study parameters

The use of PP in our ICU with or without ECMO is described in an institutional procedure. For all sessions, parameters were recorded at three time intervals: prior to prone positioning placement, after 24 h in prone position and 24 h after the return to supine position. As specified in the procedure, in order to formally objectify respiratory improvement, respiratory and VV-ECMO parameters were maintained constant throughout the entire prone positioning session. An increase in FiO_2_ and/or VV-ECMO blood flow was only considered when SpO_2_ was below 85 %. VV-ECMO and ventilator settings were adjusted according to lung recovery after the prone positioning session. Data analysis also included the recording, at the above three pre-specified times, of complete blood gas (PaO_2_, PaCO_2_, pH, HCO_3_^−^, SaO_2_), respiratory parameters (respiratory rate, Vt, PEEP, FiO_2_, pPlat, respiratory system compliance) and VV-ECMO parameters (blood flow, sweep gas flow, oxygen delivery by VV-ECMO device: FDO_2_). Respiratory system compliance (RS compliance) was computed by dividing tidal volume by pPlat (measured during an end-inspiratory pause (1 s) minus total PEEP. Total PEEP was measured by using an expiratory pause (5 s). Driving pressure was calculated as plateau pressure minus PEEP [14]. The pre-ECMO survival probability was also calculated according to the RESP score [15].

### Chest CT analysis

In patients who underwent a computed tomography (CT) scan within the 3 days prior to placement in prone position, a measurement of the amount of non-aerated lung tissue was performed according to an adapted previously-published method by Malbouisson et al. [16]. The detailed protocol is presented in the Additional file 3 (see Figure S1) [17].

### Adverse effects

Three categories were systematically reported in the procedure: (1) adverse effects related to the cannulas and VV-ECMO device during the prone position session (drop in flow necessitating fluid resuscitation, oxygenator thrombosis, cannula removal, bleeding from cannulation sites), (2) adverse effects related to the tracheal tube and the ventilator device (accidental tracheal extubation, tube displacement) and (3) adverse effects related to the other catheters (accidental wrenching of central venous or arterial lines and nasogastric tube).

### Statistical analysis

All analyses were performed using SAS software R9.3 (SAS Institute, Cary, NC, USA). The two-tailed significance level was set at *p* < 0.05. Results are respectively presented as median (1st–3rd quartiles) and frequency (percentage) for continuous and discrete variables. The paired Wilcoxon test and Fisher’s exact test were carried out for intra-group (before-after PP) and inter-group (respiratory response) comparisons, respectively. Since 11 intra-group comparisons were performed on the same subjects at the end of each session and 24 h thereafter, significance levels were adjusted for multiple testing at each respective time points. Results were analyzed for the first session (*n* = 17) as well as for all sessions (*n* = 27), considering that the increase in gas exchange of a given PP session does not predict survival [18]. Moreover, the sampling and the number of sessions did not allow for any intra-individual adjustment.

## Results

### Population description (Table 1)

Despite optimized protective mechanical ventilation and other adjuvant treatment (i.e. PP, inhaled nitric oxide, recruitment maneuvers), 44 patients received VV-ECMO during the study period for refractory acute respiratory distress syndrome. Global survival rate was 66 %. Among the latter, 17 patients underwent PP during VV-ECMO. Pre-ECMO survival probability according to the RESP score was 76 % (33–90). All of the patients had a severe ARDS according to the Berlin definition [19]. Before being placed on VV-ECMO, 13/17 (76 %) patients were prone positioned. Prior to ECMO implantation, 4/14 patients previously responded to PP in terms of oxygenation but developed major respiratory acidosis and high plateau pressure. Femoro–jugular VV-ECMO was used in 16/17 patients and femoro-femoral VV-ECMO in one patient. ARDS was related to an infectious process in all cases. Four patients presented an influenza infection at admission.

**Table 1:** Baseline characteristics

	Median (quartiles) or *n* (%), *n* = 17
Age (years)	45 (36–55)
Male gender	12/17 (71 %)
Body mass index (kg/m^2^)	27 (23–34)
SAPS II score	44 (38–59)
SOFA score (at ICU admission)	12 (8–15)
SOFA score (before first PP session)	7 (5–11)
Pre-existing conditions	
Congestive heart failure	2/17 (12 %)
Neoplasia	0/17
Chronic respiratory disorders	1/17 (6 %)
Neuropsychiatric disorders	4/17 (24 %)
None	10/17 (59 %)
Causes of ARDS	
Gram-negative pneumonia	7/17 (41 %)
Gram-positive pneumonia	8/17 (47 %)
Influenza virus	4/17 (24 %)
Abdominal septic shock	2/17 (12 %)
Sarcoidosis	1/17 (6 %)
Prone positioning before ECMO	13/17 (76 %)
Pre-ECMO survival probability (%) (RESP score)	76 (33–90)
Duration of hospitalization before ICU (days)	1 (1–2)
Duration under ECMO (days)	18 (13–26)
Duration of ICU stay (days)	54 (36–66)
Survival at discharge	16/17 (94 %)

*SAPS II* simplified acute physiology score II, *SOFA* sequential organ failure assessment, *ARDS* acute respiratory distress syndrome, *ECMO* extracorporeal membrane oxygenation, *ICU* intensive care unit

Prone positioning was considered after a median delay of 6 days of VV-ECMO (4–12). Sixteen of 27 prone position sessions were performed after this median period. Indications for PP included unsuccessful VV-ECMO weaning attempts after at least 7 days under VV-ECMO in 11/17 patients and refractory hypoxemia with PaO_2_/FiO_2_ ratio <85 mmHg despite optimal VV-ECMO and ventilator settings in 6/17 patients. For three patients (6 sessions), refractory hypoxemia was associated with elevated plateau pressure despite ultra-protective ventilation. Four patients had 3 sessions and two patients had 2 sessions. The median delay between each session was 2 days (1–4).

### Effects of prone position session on respiratory state

**Fig. 1:**
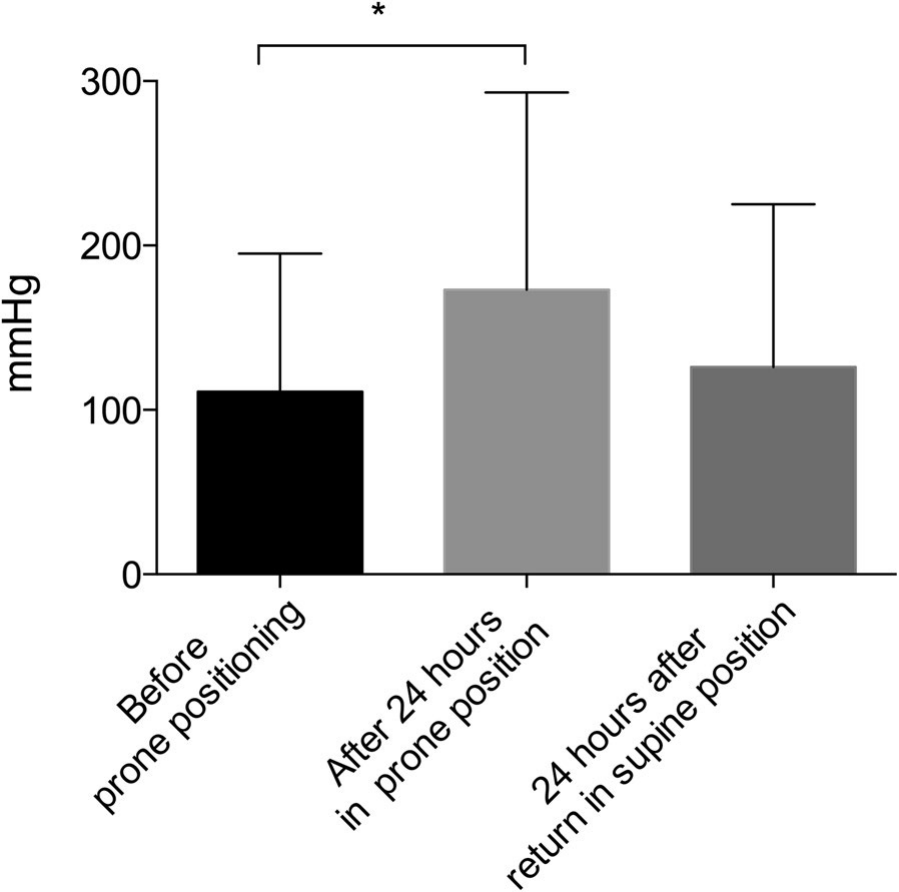
Effect of prone positioning on PaO_2_/FiO_2_ ratio before and after 24 h of prone position as well as 24 h after the return to supine position; *p < 0.05

**Fig. 2:**
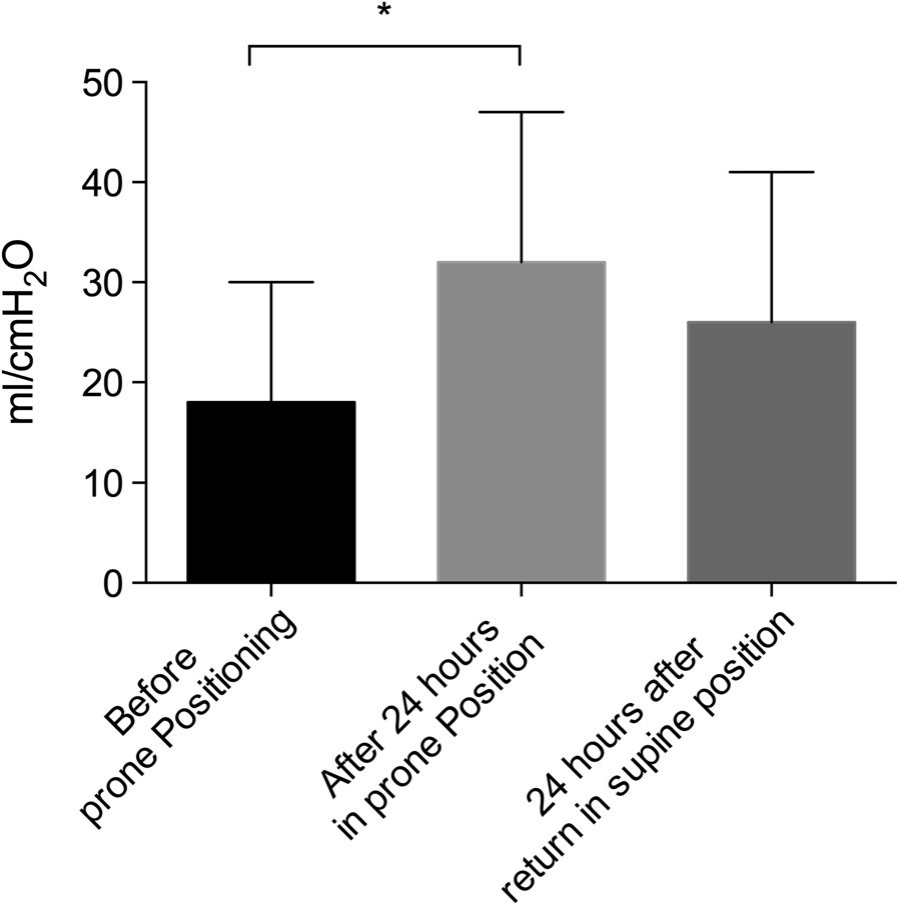
Effect of prone positioning on respiratory system compliance before and after 24 h of prone position as well as 24 h after the return to supine position; *p < 0.05

Pre-PP parameters are described in Table 2. Twenty-seven sessions were performed. All sessions had an identical duration of 24 h. In 23/27 sessions, nurses described a major increase in sputum drainage. After 24 h in PP under the same respiratory and ECMO settings, PaO_2_/FiO_2_ increased from 111 (84–128) to 173 (120–203) mmHg (*p* < 0.0001) (Fig. 1). PaO_2_/FiO_2_ increased by more than 20 % in 22/27 sessions. According to this threshold value, when PP was performed after day 7, PaO_2_/FiO_2_ ratio increased by more than 20 % in 14/14 sessions. Conversely, only 7/12 sessions had a higher than 20 % increase in PaO_2_/FiO_2_ ratio before day 7 (*p* = 0.01). RS compliance significantly increased after 24 h in PP from 18 (12–36) to 32 (15–36) ml/cmH_2_O (*p* < 0.005) (Fig. 2). Additional file 4: Figure S2 and Additional file 5: Figure S3 (see supplemental digital content) provide individual data for plateau pressure and RS compliance, respectively. Twenty-four hours after the return to supine position, tidal volume was significantly increased from 3 (2.2–4) before PP to 3.7 (2.8–5) ml/kg of predicted body weight (*p* < 0.05). Arterial PCO_2_ did not change during and after PP.

**Table 2:** Baseline characteristics

PaO_2_/FiO_2_ (mmHg)	111 (84–128)
PaCO_2_ (mmHg)	42 (39–43)
Tidal volume (ml/kg)	3.0 (2.2–4.0)
Arterial pH	7.42 (7.39–7.44)
PEEP (cmH_2_O)	12 (6–13)
Plateau pressure (cmH_2_O)	24 (22–25)
RS compliance (ml/cmH_2_O)	18 (12–36)
Respiratory frequency (cycles/min)	17 (10–25)
ECMO settings	
FiO_2_ (%)	70 (70–90)
ECMO blood flow (l/min)	4.7 (3.7–5.5)
Sweep gas (l/min)	5.0 (3.0–5.0)

*PaO*_*2*_ *, PaO*_*2*_ arterial oxygen pressure, *PaCO*_*2*_ partial pressure of carbon dioxide in arterial blood, *FiO*_*2*_ fraction of expired oxygen, *PEEP* positive end expiratory pressure, *RS compliance* respiratory system compliance, *ECMO* extracorporeal membrane oxygenation

### Correlation between lung condensation and PaO_2_/FiO_2_ response

Fifteen CT scans were performed prior to placement in prone position. All patients exhibited a high percentage of non-aerated or poorly-aerated lung parenchyma prior to PP: 52 % (41–62). Thus, no correlation was found between the increase in PaO_2_/FiO_2_ ratio and the amount of non-aerated lung tissue measured on chest scans (*r* = 0.064, *p* = 0.82).

### Adverse events attributable to PP

In one patient, an oxygenator thrombosis occurred while one other patient required fluid resuscitation in order to correct a drop in VV-ECMO flow.

No adverse event was related to either the tracheal tube, the ventilator device or other catheters.

## Discussion

The main findings of the present study are that (1) in a majority of patients, PP markedly improved oxygenation during VV-ECMO and was not associated with side effects confirming the Guervilly et al. study, (2) the improvement in oxygenation appeared to be more efficient when applied after 7 days of VV-ECMO, (3) contrary to the Guervilly et al. study, PP was associated with an improvement in lung compliance therefore allowing the use of increased Vt and (4) there was no correlation between the volume of condensation and PP efficiency.

### Prone positioning during VV-ECMO improves oxygenation

All currently published studies are consistent with regard to the effect of PP on oxygenation during VV-ECMO. Clearly, PP improves oxygenation in a majority of patients. Moreover, previous studies mainly studied the effects of PP when performed late after VV-ECMO initiation (8 days in the Guervilly et al. study). It appears that for some patients PP was effective under ECMO and ineffective prior to ECMO. It is likely that time is an important factor in allowing lung healing and symptomatic treatments such as antibiotics to be effective. The decrease in lung aggression secondary to ultraprotective ventilation should also be considered.

### Prone positioning during VV-ECMO improves respiratory system compliance and allows increased tidal volume

Importantly, PP was associated with a marked decrease in plateau pressure in patients in whom it was not possible to maintain this pressure under 25 cmH_2_O. In randomized controlled trials comparing supine and prone positions, the results on respiratory compliance have been inconsistent. Indeed, Mancebo et al. noted higher compliance of the respiratory system in the prone position, whereas Guerin et al. and Taccone et al. did not [20, 21]. Previous studies using PP during VV-ECMO also failed to demonstrate any significant changes in static compliance. Guervilly et al. suggested that the improvement in oxygenation without concomitant increase in respiratory static compliance and without decrease in PaCO_2_ at constant levels of minute ventilation and sweep gas flow do not suggest lung recruitment by PP with relatively small Vt and high levels of PEEP but rather an improvement in VA/Q mismatch. Unfortunately, no CT scan data were provided in their study. Herein, all of our patients had major posterior condensation (>50 % of lung volume) and thus it is likely that PP in this situation allows lung recruitment. Arguing in favor of this hypothesis is the fact that PP further allows the use of increased Vt. Importantly, in 23/27 sessions, nurses described a major increase in sputum drainage that may have contributed to recruitment. Another hypothesis to explain this discrepancy is that prolonged PP sessions were used in our study (24 h as opposed to 12 h), which may have contributed to the observed improvement in compliance. Finally, the discrepancy could be due to different patient populations, i.e. patients still under sedation in whom it was not possible to use partial mode of ventilatory support or to a different phase of the disease comparatively to previous reports.

The driving pressure (plateau pressure minus PEEP) is associated with ARDS prognosis, [14] but also with treatment efficiency when associated with decreases in Δ*P*. Interestingly, the driving pressure observed in our study was relatively low and the use of PP was associated with a further decrease in driving pressure.

### Study limitations

Although the present constitutes the largest study relative to the use of PP during VV-ECMO, the number of studied patients remains relatively small. Nevertheless, severe ARDS treated with ECMO and needing prone positioning is a rare occurrence and thus it is unlikely that a multicenter randomized study will be performed. Accordingly, only 36 cases have been previously reported in the literature in which session durations differed considerably. Therefore, we firmly believe that this study adds valuable information, first in confirming previous but scarce data but also in extending these data with new findings, notably on compliance.

The small sample size may also have had a bearing on our findings regarding the comparison between early and late sessions. In addition, specific predefined criteria were chosen as to whether or not to use PP in our VV-ECMO patients. Therefore, our results and conclusions are only valuable when considering the present clinical algorithm. Finally, because of the retrospective nature of the present study, it was not possible to study the mechanism involved in PP effects during VV-ECMO.

### Conclusions

When used in combination with VV-ECMO, 24 h of prone positioning improves both oxygenation and respiratory system compliance. Moreover, our study confirms the absence of serious adverse events. Currently, it remains unknown when and for which indication prone positioning should be systematically applied during VV-ECMO. Therefore, further studies are needed to better delineate prone positioning indications during VV-ECMO.

## Additional files

10.1186/s13613-015-0078-4 Protocol for prone positioning under VV-ECMO (Supplemental Digital Content movie).
10.1186/s13613-015-0078-4 Protocol for prone positioning under VV-ECMO (Supplemental Digital Content movie).
10.1186/s13613-015-0078-4 CT scan analysis. In a first step in the analysis of CT images (A), lung contours were manually delineated from chest wall, mediastinum and pleural effusion. This resulted in the crude lung volume (B). (Actual, True) lung volume (C) was obtained by excluding large vessels (densities above 150 HU on enhanced scans) and bronchi lumen and hyperinflated lungs (densities below 900 HU). Lastly, a normally-aerated lung was defined as having a density below 100 HU (D).
10.1186/s13613-015-0078-4 Evolution of plateau pressure before and 24 h after prone positioning. Individual data.
10.1186/s13613-015-0078-4 Evolution of respiratory system compliance before and 24 h after prone positioning. Individual data.
