# Supplementary material for: Prolonged prone positioning under VV-ECMO is safe and improves oxygenation and respiratory compliance
Source: Ann Intensive Care. 2015 Nov 4;5:35. doi: 10.1186/s13613-015-0078-4 (PMC4633431; doi:10.1186/s13613-015-0078-4)
Supplement: Supplementary file 2 — 10.1186/s13613-015-0078-4 Protocol for prone positioning under VV-ECMO (Supplemental Digital Content movie). [file 13613_2015_78_MOESM5_ESM.docx]

**Supplementary material**

**Protocol for prone positioning under VV-ECMO (Supplemental Digital Content movie)**. Briefly, the patient was sedated and paralyzed as described above. Eight staff members were required for the procedure. One person was dedicated to the management of the upper airways, one to the management of the VV-ECMO lines and one to set a new bed sheet and specific pillows. The caregiver in charge of VV-ECMO at the foot of the bed had to coordinate the team. The four other persons stood at each side of the bed. A critical care physician was systematically present in the room. Specific attention was given to the following items: 1) the direction of rotation (to the left or to the right) was decided giving priority to the reinjection VV-ECMO line (most often cannulated in the right internal jugular vein); 2) the person dedicated to the VV-ECMO lines continuously controlled the VV-ECMO flow, the integrity of the lines (no kinking) and the integrity of the dressing covering the lines; 3) A specific triangular pillow was placed under the anterior iliac crests to avoid any compression of the femoral cannula.

**Chest CT analysis**. . CT realization was driven by the patient’s condition (patient under VV-ECMO and apneic ventilation); the procedure was performed during the expiration period, using thin sections (1.2 mm to 2.0 mm), after intravenous injection of contrast material except in 2 cases. Images were reconstructed with a dedicated chest or lung algorithm. Image analysis was performed on a dedicated workstation (Volume Viewer version 7.9.1, General Electric Healthcare, Milkaukee, IL, USA) by a single observer, blinded to the clinical data; magnification and windowing were adjusted when deemed appropriate. In order to measure the volumes of non- and poorly-aerated lung parenchyma at the same PEEP condition before the prone positioning session, the following method was used (Additional figure 1):

1. Using a mediastinal-adjusted windowing, the observer manually delineated the right and left lung parenchyma from pleural effusion, chest wall and mediastinal structures on each slice; the sum of each parenchymal section defined the crude lung volume (Supplemental Digital Content figure 1, panels A and B);
2. A second step aimed at obtaining the real lung volume (Additional figure 1, panel C) by removing both:
   1. large vessels, characterized by a CT attenuation ranging above 150HU, and
   2. large bronchi, i.e. air, characterized by attenuation below -900HU; this also removed areas of hyperinflated lungs, such as emphysema .
3. Finally, the poorly and non-aerated lung areas, i.e. pulmonary condensation, were separated from the exploitable lung volumes, i.e. the aerated lung areas, by using a threshold of -100HU (Supplemental Digital Content figure 1, panel D) [17].
