# Supplementary figures and images for: Prolonged prone positioning under VV-ECMO is safe and improves oxygenation and respiratory compliance
Source: Ann Intensive Care. 2015 Nov 4;5:35. doi: 10.1186/s13613-015-0078-4 (PMC4633431; doi:10.1186/s13613-015-0078-4)

## Slide 1
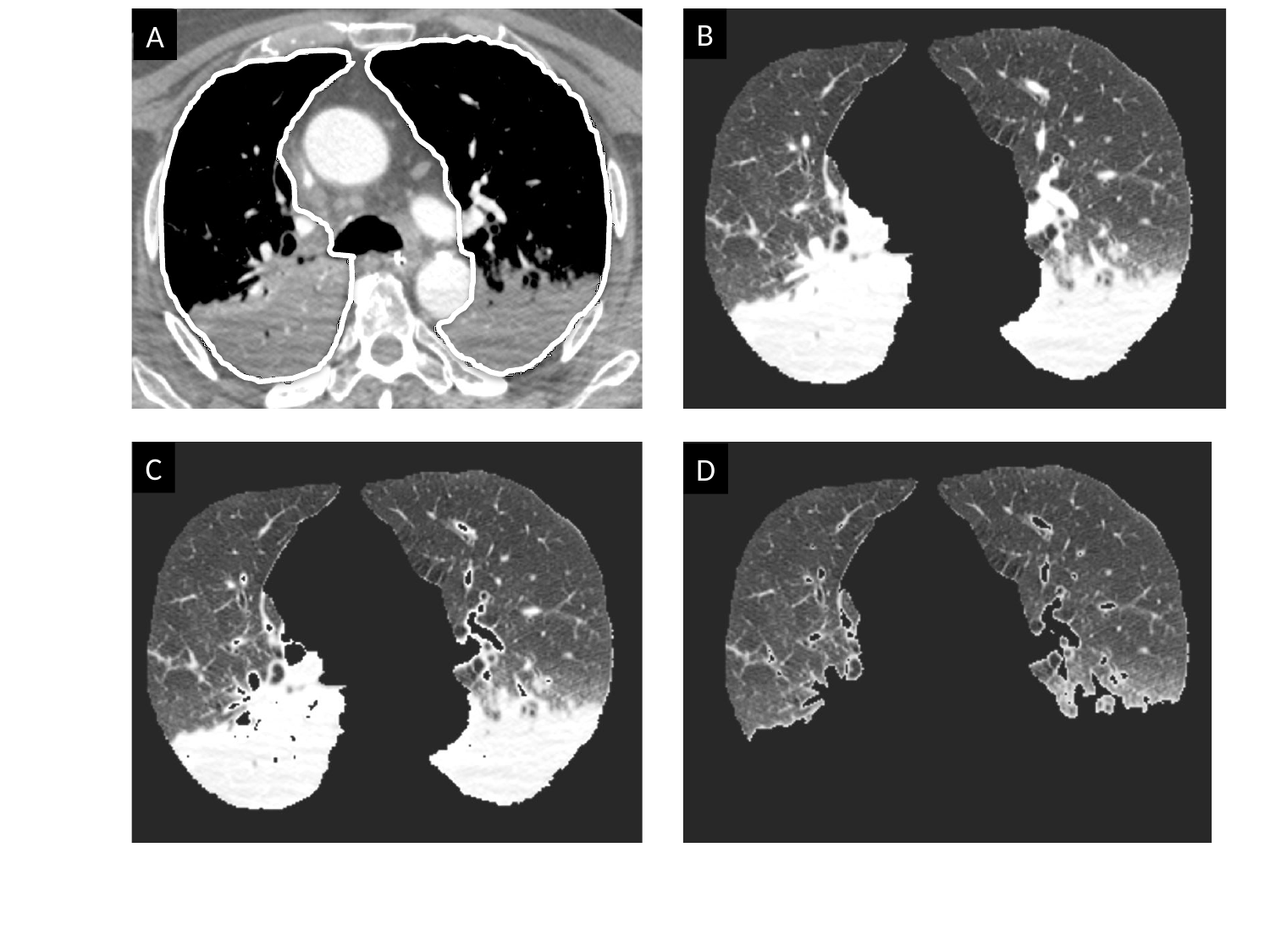

B
A
C
D

Supplement: Supplementary file 3 — 10.1186/s13613-015-0078-4 CT scan analysis. In a first step in the analysis of CT images (A), lung contours were manually delineated from chest wall, mediastinum and pleural effusion. This resulted in the crude lung volume (B). (Actual, True) lung volume (C) was obtained by excluding large vessels (densities above 150 HU on enhanced scans) and bronchi lumen and hyperinflated lungs (densities below 900 HU). Lastly, a normally-aerated lung was defined as having a density below 100 HU (D). [file 13613_2015_78_MOESM1_ESM.pptx]

Plateau Pressure  
cmH<sub>2</sub>O

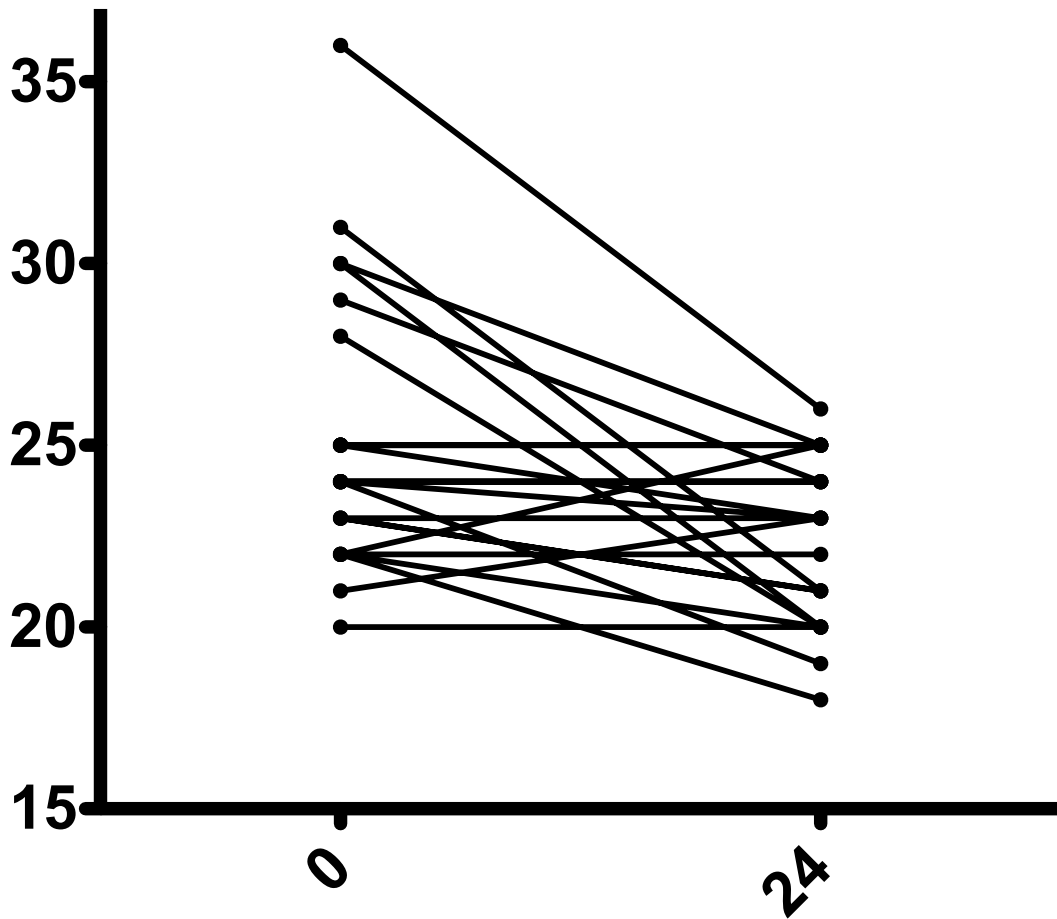

Supplement: Supplementary file 4 — 10.1186/s13613-015-0078-4 Evolution of plateau pressure before and 24 h after prone positioning. Individual data. [file 13613_2015_78_MOESM2_ESM.pdf]

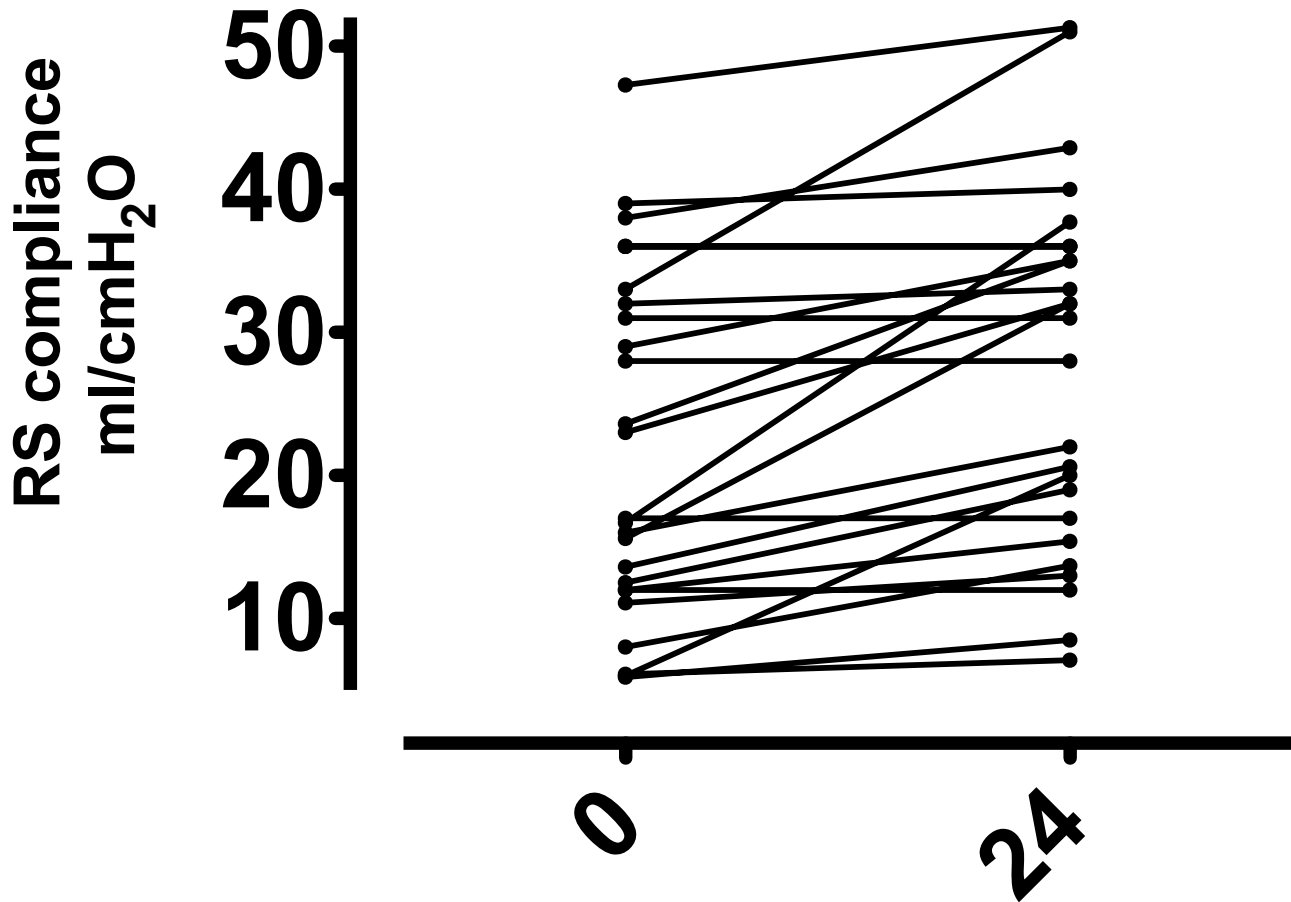

Supplement: Supplementary file 5 — 10.1186/s13613-015-0078-4 Evolution of respiratory system compliance before and 24 h after prone positioning. Individual data. [file 13613_2015_78_MOESM3_ESM.pdf]
